# A Dual Vibrational Probe Framework for Studying Solvation in Highly Heterogeneous Molecular Environments

**DOI:** 10.1021/acs.jpclett.6c02383

**Published:** 2026-10-14

**Authors:** Samadhan H. Deshmukh, Laura X. Sepulveda-Montaño, Daniel G. Kuroda

**Affiliations:** Department of Chemistry, 5779 Louisiana State University, Baton Rouge, Louisiana 70803, United States

## Abstract

Vibrational probes are essential for studying interactions and dynamics of complex environments, but they often rely on computer simulations to describe the system. Here, a dual-IR-probe approach based on the alkyne molecular framework is demonstrated for two probes: MOC (low polarity) and TFPB (nonpolar). In these dual-probes, the secondary IR-active group allows their precise localization, while the alkyne group is used to probe the dynamics. The approach is validated in two complex liquids: a deep eutectic solvent (DES) and a water micelle. In the DES, the dual-IR scheme experimentally locates MOC in one specific domain. In water micelles, MOC and TFPB localize selectively at the interface and within the hydrophobic core, respectively, providing an independent characterization of the two environments. The results demonstrate that the alkyne-based dual-probe is a versatile molecular framework for characterizing the structure, interactions, and dynamics of complex liquids using only experimental methods.

## Graphical abstract

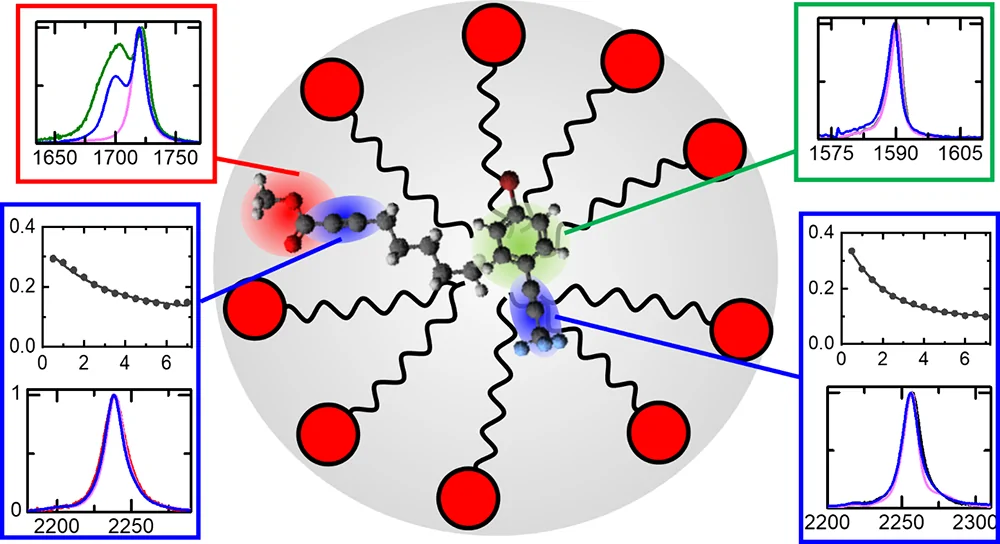

Vibrational spectroscopy, through the use of ultrafast laser methods, has become a powerful and robust tool for the molecular characterization of a variety of complex systems, such as deep eutectic solvents.^1–3^ IR spectroscopy uses vibrational modes of molecules because their IR absorption bands contain metrics, such as central frequencies, intensities, and lineshapes, which directly reflect local interactions and dynamics. Consequently, vibrational spectroscopy has been extensively used to investigate liquids, biological systems, and materials providing key insights into interactions, dynamics, and structure–function relationships.^4–8^ Many molecules contain suitable vibrational modes for studying molecular systems, but these modes often appear in congested regions of the spectrum, making it difficult to disentangle their responses from those of other molecules. In these cases, exogenous IR vibrational probes are used because they allow the selection of an appropriate vibrational mode to avoid such congestion and overlap.^9^ Moreover, in many cases, it is possible to select appropriate probes having vibrational lifetimes that are larger than those found in the modes of the molecular components of the sample.^10–12^ Various IR probes have been used to study the interactions and dynamics in numerous complex systems, including the nitrile, carbonyl, azide, carbon/oxygen-deuterium, thiol, phosphate, and fluorocarbon groups.^13,14^ Hence, IR probes have been widely used in various molecular contexts as a spectroscopic window for studying complex systems.^9,15^

The use of vibrational probes in highly heterogeneous molecular environments offers a unique opportunity to obtain molecular information, such as dynamics and interactions. However, this molecular information is usually difficult to interpret because it could be related to many distinct local structures and dynamical processes.^16^ For example, a vibrational probe in a highly heterogeneous molecular system may experience a wide variety of interactions, such as ionic, hydrogen bonds, as well as a distribution of different solvation environments, all contributing to frequency shifts, line widths, and spectral diffusion. This mixed contribution makes it very difficult to rationalize the molecular system from experiments alone.^17,18^ To understand site-specific interactions in complex environments, experiments are usually complemented with molecular dynamics (MD) simulations.^8,19–22^ However, the use of MD simulations has its own limitations as it heavily relies on a suitable representation of the molecular system (i.e., a good force field), which often requires the model to be validated prior to its use.^23,24^ Consequently, the applicability of the experimental-MD approach is often limited in complex environments, where force-field validation is difficult to perform.

The interpretation of IR spectroscopic metrics from vibrational probes can be simplified using a dual-IR-probe. The dual-IR-probe strategy consists of selecting a molecular system with two vibrational reporters, such that they provide complementary information. By including redundancy, this strategy enables a more direct and intuitive characterization of complex molecular environments.^25^

Hence, different aspects of the system, including interactions and dynamics, can be probed because the appropriately selected IR-active moieties respond selectively to specific local environments. This strategy reduces ambiguity and allows direct mechanistic interpretations. The dual-IR-probe strategy has previously been used to investigate complex systems, including RNA nucleosides, DNA strands, cytoplasm-like environments, and proteins, where the probes have been used to report on vibrational coupling or a shared local environment.^25–28^ In contrast, the probes used in the present study do not exhibit appreciable vibrational coupling and preferentially partition into distinct microenvironments. The lack of significant coupling allows us to obtain complementary information about the system from the two vibrational modes independently.

One of the simplest examples of a dual-probe in the same molecule is semideuterated water (HDO), as this molecule reports on the chemical environment through both the bending and stretching normal modes.^29–33^ Similarly, other functional groups have two intrinsic vibrational modes, such as carbonyl and O–H stretches in carboxylic acids,^34^ amide-I and NH stretch in amides,^35,36^ the ring breathing and nitrile stretch modes of benzonitriles,^37^ and the symmetric and asymmetric P-(OH)_2_ and PO_2_^–^ stretch vibrations in phosphates,^38,39^ and have been used in a dual-probe scheme to retrieve molecular information about the system. Another approach consists of including two selected vibrational probes, such as azido and nitrile groups,^25,28^ in the same molecule. However, due to the need for an active vibrational mode, all the aforementioned dual-IR probes contained polar terminal groups. This results in systems having an affinity for polar and hydrophilic chemical environments, which cannot be easily modified. Moreover, the large majority of these dual-probe systems tested similar properties of the molecular environment because the two vibrational model involve the shared atoms, such as the case of O–H stretch and H–O-D bending modes of water. However, dual vibrational probes with the two groups probing different properties of the environment and with a polarity selected to study distinct molecular environments, from hydrophilic to hydrophobic, have not been addressed.

Among well-known IR active moieties, the carbon–carbon triple bond (alkyne) is uniquely positioned for implementing the proposed dual-IR-probe approach, since it is not a terminal functional group, is sensitive to its molecular environment, and appears in the spectrally transparent IR window between 2000 and 2300 cm^–1^.^3,9,40–43^ In addition, the absence of lone electron pairs and heteroatoms in the carbon triple bond results in very weak directional interactions, such as the O–H•••π interactions,^44–46^ making the alkyne stretch a suitable probe for studying pure solvation dynamics.^3,40–42^ However, symmetric alkyl systems (R–C≡C–R) have a weak or non-IR-active C≡C stretch due to their small IR transition dipole.^40,42,47,48^ This has made alkynes good probes in Raman because of their large Raman cross sections, site specificity, and solvatochromism.^49–52^

Despite these shortcomings, the alkyne group is an excellent molecular framework for the dual-IR-probe approach because the careful selection of its substituents not only enhances the inherently weak alkyne transition dipole by breaking its symmetry,^40–43^ but also determines the overall polarity of the molecule. For example, positioning a carbon triple bond between a carbonyl group and a long chain alkyl group substantially increases its molar extinction coefficient and overall polarity. This makes the alkyne stretch a suitable framework for creating dual-IR probes with polarity that can be tailored to investigate different regions of heterogeneous liquids. Hence, the alkyne functional group has potential for its widespread application in dual IR probes, but the dual-probe functionalization of alkyne group has not been realized yet. Further, the dual-probe has not been implemented for a molecular probe having two spectrally well-resolved and sufficiently intense IR-active vibrational modes, and an overall polarity (from nonpolar to polar) defined by the substituents, which can be easily attained using the alkyne framework.

Here, the dual-probe strategy is used for internal alkynes. By systematically changing the terminal methyl group of an alkyne with different IR-active moieties, a significant enhancement of the alkyne transition dipole is observed. This effect is well described by the series: 2-octyne (OTY), 2-octyne-1-ol (OTO), methyl octyne carbonate (MOC), and 1-bromo-4-(3,3,3-trifluoroprop-1-yn-1-yl)­benzene (TFPB) (Figure 1A). Importantly, the last three molecules have well-known IR active and solvatochromic groups: hydroxyl (−OH),^53,54^ ester (−CO_2_-),^55^ and phenyl (-Ph).^21,56,57^ While all the molecules in this series show the expected alkyne stretch region (2100–2300 cm^–1^), a notable increase in the molar absorptivity of the C≡C stretch is observed (Figure 1B) from the ∼1 cm^–1^ M^–1^ in the quasi symmetric R-CH_2_–C≡C–CH_3_ structure of OTY to ∼ 650 cm^–1^ M^–1^ in the completely asymmetrically substituted alkyne group of TFPB. A secondary small peak is also observed in the alkyne region (Figure 1B) due to the presence of a Fermi resonance, which is sensitive to both alkyne substituents and solvent (Figures S1 and S2).^58,59^ Notably, MOC and TFPB exhibit much stronger absorptivities than OTY and OTO, and are comparable to previous alkyne-based probes.^40,41^ The TFPB case is notable because the use of the strongly electron-withdrawing trifluoromethyl (−CF_3_) and hydrophobic phenyl groups not only induces a strong asymmetry to the alkyne stretch, which results in a large transition dipole, but also produces a hydrophobic molecule.^60^ While OTY and OTO have an inherent dual-probe character due to their O–H and C–H­(D) stretches, respectively, the relatively low transition dipole moment of their alkyne stretch limits their applicability and therefore, they were not further explored in this work.

**Figure 1:**
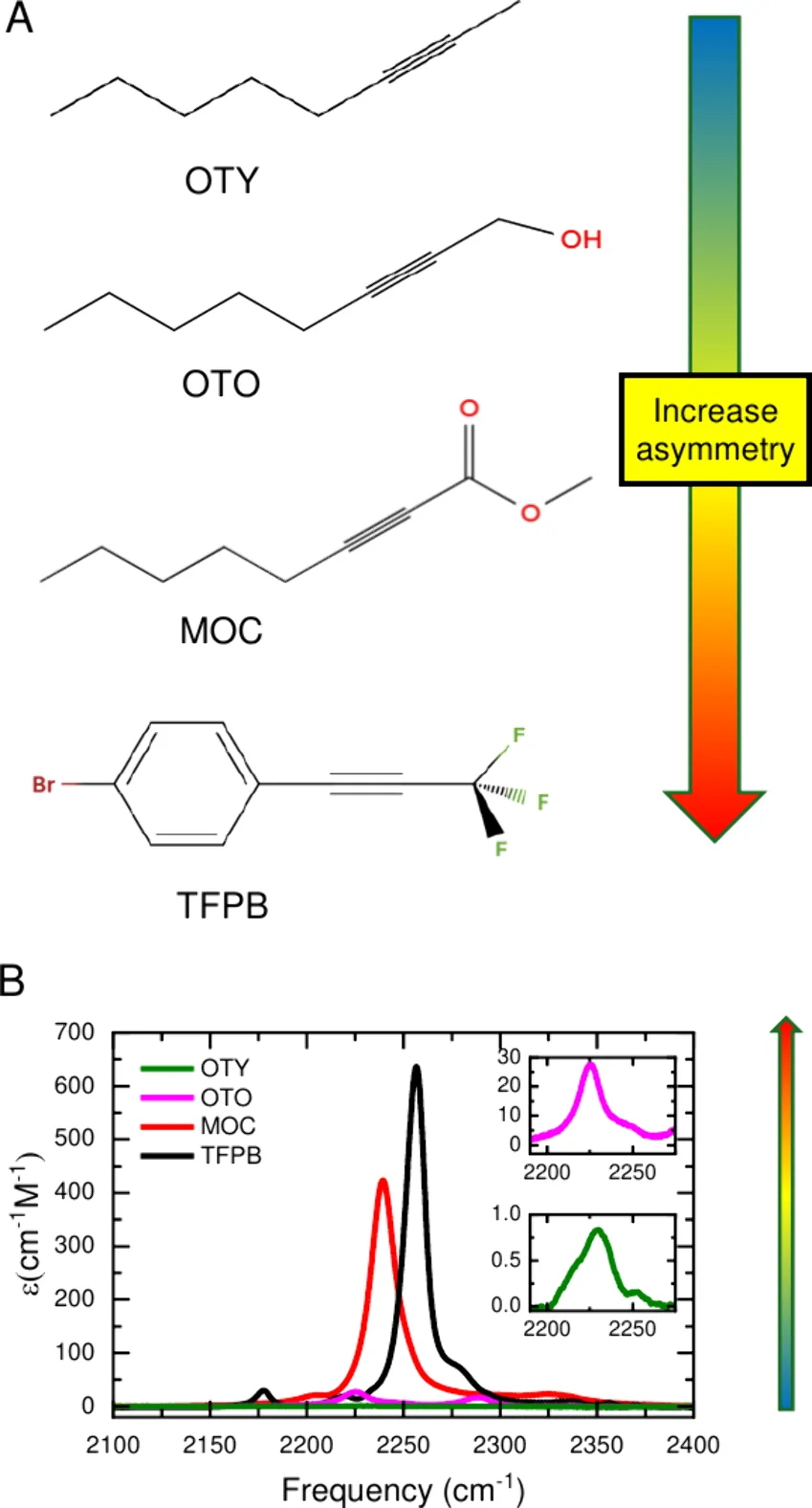
Panel A shows the chemical structure of the different alkyne IR probes, while panel B shows the molar extinction spectra of OTY (green), OTO (pink), MOC (red) and TFPB (black) in carbon tetra chloride (CTC). The two insets show the alkyne stretch of both OTY (green) and OTO (pink) scaled to the same size.

The suitability of the dual-probe nature of MOC and TFPB was first examined by investigating the solvatochromism of their IR-active groups. The IR spectra of the alkyne group in various solvents (Figure 2 and Figure S2) show that their C≡C stretches appear at 2200–2300 cm^–1^ for MOC and TFPB. The second IR reporter in MOC and TFPB appears at much lower frequencies: the carbonyl stretch at 1650–1780 cm^–1^ and the ring mode at 1570–1600 cm^–1^ (Figure 2) in agreement with previous reports.^21,57,61^ The solvatochromism of the alkyne stretch in both polar and nonpolar solvents (Tables S1 and S2) is modest for the two probes (∼8 cm^–1^ for MOC and ∼4.5 cm^–1^ for TFPB, Figure 2) and is consistent with a previous study.^40^ Interestingly, the solvatochromic behavior of the carbon–carbon triple bond differs from that of other IR active triple-bond groups, such as benzonitriles, where aprotic solvents like DMSO induce a red shift and methanol a blue shift.^62,63^ Hence, the solvatochromic behavior of these alkyne stretches cannot be explained solely by solvent parameters such as dielectric constant and Kamlet–Taft hydrogen-bond donating (α) and accepting (β) abilities (Figure S3),^64^ but correlates with the solvent polarizability (π*) in both MOC (Pearson correlation coefficient R = 0.964) and TFPB (R = 0.637). While MOC has a strong linear dependence, TFPB exhibits a significantly weaker correlation and a smaller overall frequency shift, likely due to steric and electronic effects that lower its sensitivity to bulk solvent polarity.

The solvatochromic correlations of the alkyne stretch indicate that nonspecific dispersive and inductive interactions dominate the frequency shift of the group due to the lack of strong directional interactions, such as hydrogen bonds. For example, this effect is observed in the methanol sample, where the carbon triple bond solvatochromism falls within the expected linear behavior. Moreover, DFT computations reproduce the solvatochromic behavior observed for the two modes of MOC in an implicit dielectric continuum medium (Figure S4) in agreement with the experiment. These results suggest that dispersion interactions play a significant role in the solvatochromism of this dual probe. It is also possible that the presence of Fermi resonances affects the solvatochromism results, but the similar solvatochromic behavior reproduced by harmonic frequency calculations makes this possibility unlikely. However, the molecular origin of this behavior will be addressed in a future study.

The observed solvatochromism correlation also extends to the carbonyl stretch of MOC and the ring breathing mode of TFPB as they also present strong correlation with π* (R_CO_ = 0.978 and R_RM_ = 0.914 in Figure 2). The high correlation of the vibrational frequency with solvent polarizability (π*) for both modes in either probe shows that the solvent polarizability serves as a useful metric for describing the molecular environment observed by either probe.

**Figure 2:**
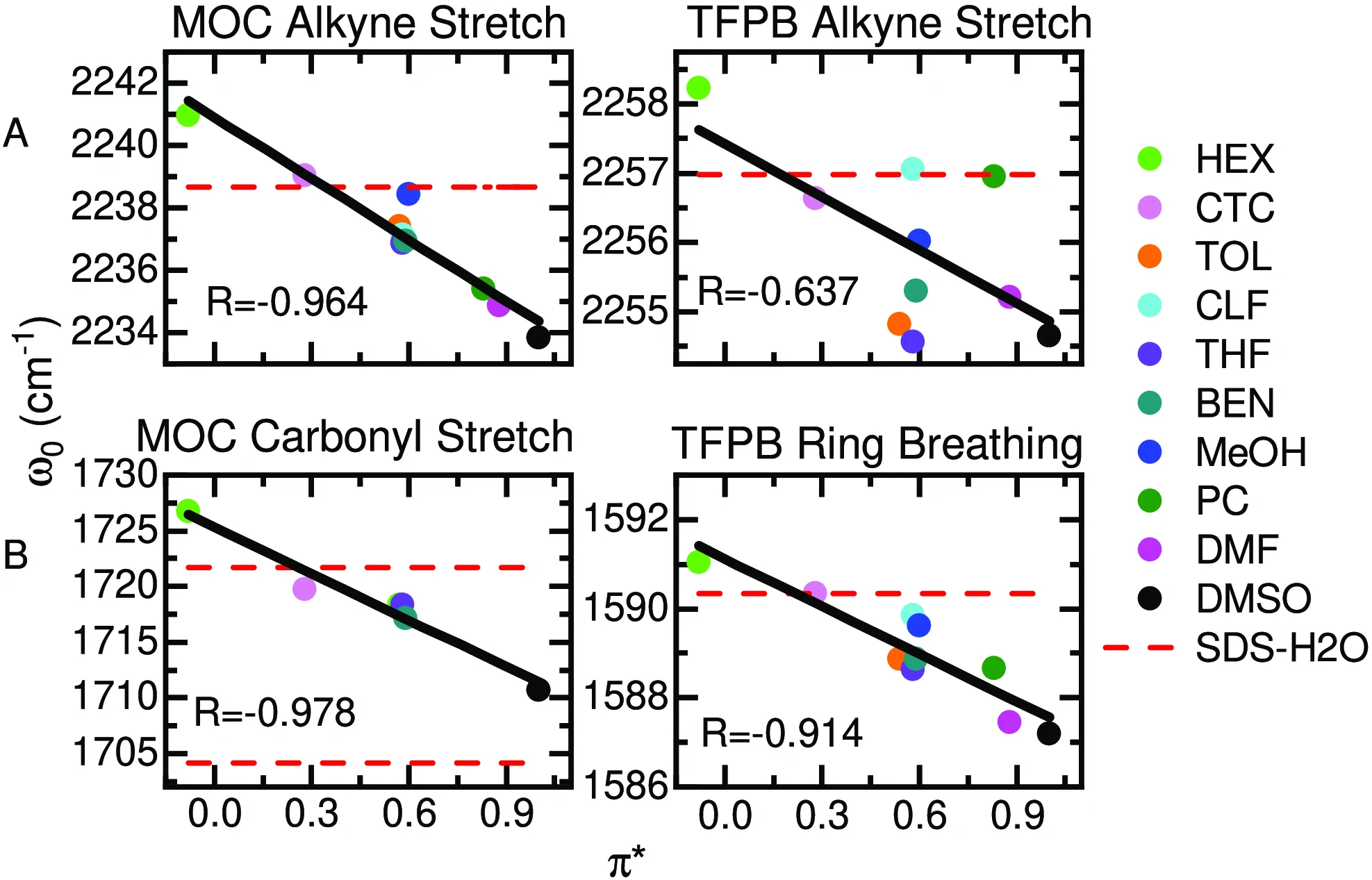
Solvatochromism of the two dual probe molecules in various solvents versus solvent polarizability (π*). The first and second columns correspond to the probe frequencies for MOC and TFPB, respectively. Panel A corresponds to the alkyne stretch, while panel B corresponds to the secondary modes, carbonyl stretch for MOC and ring mode for TFPB. R represents the Pearson correlation coefficient.

The two vibrational modes of the probes offer complementary information on the heterogeneous environment, but serve distinct roles. In these cases, the secondary mode of the probe provides complementary information that helps identify and confirm the local environment, and the isolated alkyne stretch serves as the dynamics reporter of the molecular environment. Thus, this dual-probe strategy combines complementary information for the environment characterization with dynamical information obtained from the isolated alkyne vibrational mode.

Having established that the two vibrational reporters respond consistently to changes in the local environment, it remains to be demonstrated whether the alkyne reporter can also provide information on solvation dynamics. The suitability of the dual-IR-probe for studying the dynamics of the system is demonstrated by measuring the spectral diffusion dynamics using 2DIR spectroscopy. Note that both the carbonyl stretch of esters and the phenyl ring mode have been previous described in the literature.^21,65^ To this end, spectral diffusion reported by the alkyne stretch of MOC and TFPB in MeOH and DMSO was investigated. The solvation dynamics derived from the time evolution of 2DIR spectra (Figure S5) show the typical picosecond time scale of local motions in organic solvents.^53^ The characteristic times of the FFCFs, obtained by fitting with a single-exponential decay of the form: *FFCF*(*T_W_*)=*A_1_e* ^–*t*/τ^+*A*_2_ (see Figure 3), indicate that MOC has very similar FFCF correlation times in both MeOH (2.2 ± 0.1 ps) and DMSO (2.4 ± 0.1 ps) and these values are comparable to the dynamics observed in the complex DES environment, where a dynamics with a characteristic time of 2.9 ± 0.2 ps was observed.^3^ The small changes in the dynamics as a function of the solvent are indicative of local dynamics being controlled by an internal mode, as previously seen for carboxylates and amides.^66,67^ However, the amplitudes of the FFCF short (A_1_) and long (A_2_) time scales show statistically different values (Table S3), agreeing with previous results, in which the alkyne group of MOC senses the pure dynamics of the molecular environment.^3^ In contrast, 2DIR measurements of the TFPB probe show sensitivity to the solvent in the dynamics, as the correlation times become 1.6 ± 0.1 ps for MeOH solution and 3.0 ± 0.2 ps for DMSO (Table S4). Interestingly, both probes have different inhomogeneous contributions (CLS at Tw = 0 ps)^22^ in the two solvents, with the alkyne group in DMSO exhibiting higher inhomogeneity than in MeOH, in agreement with a local mode controlling both solvent dynamics and vibrational couplings. Further, the solvent dynamics observed by TFPB in MeOH and DMSO are similar to that of methyl thiocyanate (MeSCN), suggesting that TFPB captures comparable aspects of the underlying solvation dynamics in these two solvents.^22^

**Figure 3:**
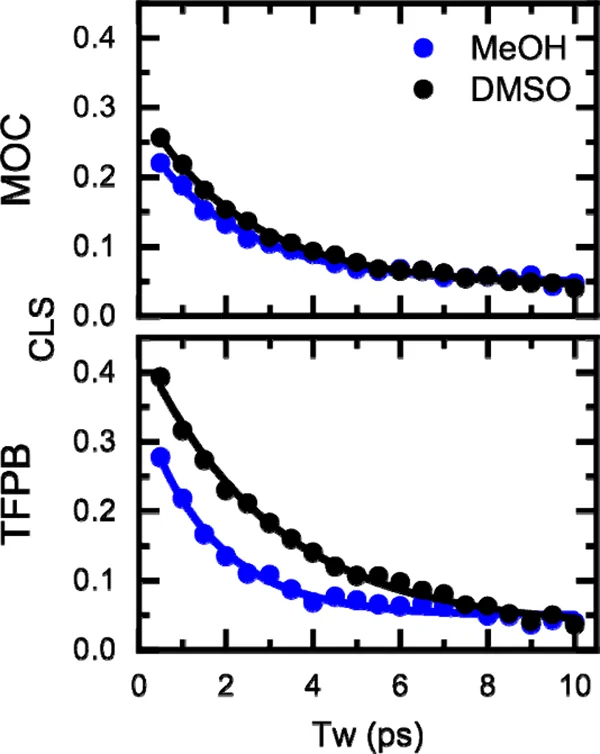
Center line slope (CLS) as a function of the waiting time (T_W_) of MOC (top panel) and TFPB (bottom panel). The solvents used were MeOH (blue) and DMSO (black).

The first example demonstrating the utility of the dual IR probe scheme in molecularly heterogeneous systems is presented for MOC in a deep eutectic solvent (DES) formed by lauric acid (LA) and *N*-methylacetamide (NMA) at a 1:4 molar ratio.^1^ This NMA-LA DES is particularly challenging to characterize using vibrational probes because it is highly heterogeneous due to the presence of interdigitated LA domains embedded in a continuous NMA domain.^1,7,68^ Indeed, previous studies have shown the intricacies of localizing IR probes, such as W­(CO)_6_,^7^ chloroform,^68^ and MOC,^3^ in the NMA-LA DES. In particular, the low solvatochromic changes exhibited by MOC when switching from the molecular environment of pure NMA to pure LA (Figure 4) has made the assignment of its DES location highly problematic, as it requires extensive and rigorous IR line shape analysis.^3^ However, the second vibrational mode of MOC (i.e., the carbonyl stretch) provides a robust assignment of probe localization using FTIR measurements alone.

**Figure 4:**
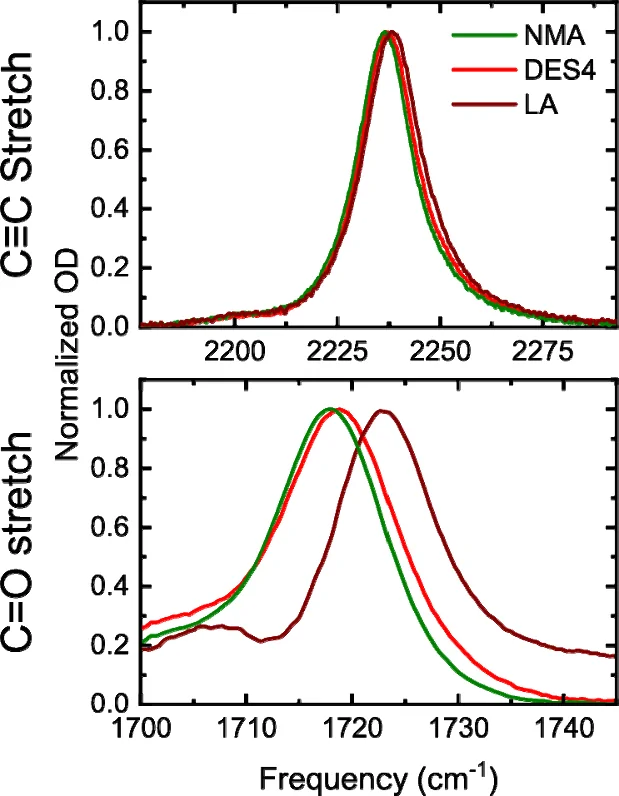
Normalized FTIR spectra of MOC in LA (brown), NMA (green) and LA:NMA in 1:4 (DES4 red) molar ratio for the alkyne stretch in the top panel, reprinted with permission from ref 3 copyright (2024) AIP Publishing, and for the carbonyl stretch in the bottom panel.

Contrary to the small solvatochromism of the MOC alkyne stretch (Figure 4), the MOC carbonyl stretch, appearing in the 1700 to 1740 cm^–1^ region, shows pronounce solvent-dependent shift of its central frequency. A comparison of the MOC carbonyl stretch in the DES with the corresponding spectra in pure LA or pure NMA is sufficient to assess the location of the probe in the complex environment due to its close resemblance to pure NMA. The result strongly support MOC residing within the NMA domains of the LA-NMA DES, confirming previous results using only the alkyne stretch.^3^ This result reinforces the utility of the dual probe approach for localizing the probe in a complex environment, such as that produced by a nonionic DES.

The dual-probe strategy of both MOC and TFPB is further illustrated using aqueous sodium dodecyl sulfate (SDS) micelles. The SDS micelle system is particularly interesting because it contains three distinct microenvironments (i.e., the hydrophobic core, interface, and water pool) where the probes can be located. Based on the polarities of the probes, the amphiphilic MOC and the hydrophobic TFPB are expected to be located at the interface and within the hydrophobic core, respectively.

FTIR spectra of MOC in SDS micelles (Figure 5) reveal that its alkyne stretch band is centered at 2239 cm^–1^, which according to the solvatochromic model corresponds to a polarity (π*) of 0.34. This value is close to that of carbon tetrachloride (π*­(CTC) = 0.28), indicating that the alkyne stretch of MOC senses a nonpolar environment. While this result could suggest that MOC is located in the hydrophobic core of the micelle, the solvatochromism is too small to unambiguously locate the probe, as the alkyne stretch of MOC in MeOH presents a similar central frequency of 2238 cm^–1^ (Figure 5). However, the second vibrational reporter of MOC, the carbonyl stretch, reveals the presence of two bands. Previous 2DIR spectroscopic studies of methyl acetate in water and methanol rule out the possibility that these two bands originate from syn/anti conformational equilibria,^65,69^ (Figure 5) and instead, show that they indicate the existence of two distinct species, where the low frequency carbonyl band corresponds to a hydrogen-bonded state, and the high frequency peak to its non-hydrogen bonded counterpart.^65,70,71^ Hence, the MOC carbonyl stretch locates the probe in an environment where it can have both hydrogen bonded and non-hydrogen bonded states. The hydrogen bonded states of MOC carbonyl combined with the nonpolar environment perceived by its carbon triple bond positions the probe at the interface. It is also possible that MOC coexists between a fully hydrated state and one at the micelle interface. However, this possibility is not considered due to the poor solubility of the probe in water and the absence of a MOC state having two hydrogen bonded water molecules, which is only observed in the carbonyl region when the ester group is fully solvated by water.^70^ Furthermore, concentration-dependent studies in both MeOH and hexane (Figure S7) do not show changes in line shape making it unlikely that the differences in the spectroscopic signatures arise from aggregation. To further confirm the proposed location of MOC in the micelle, the IR spectrum of the probe in SDS is compared with those obtained in CTC and MeOH. As expected, the spectrum of the carbonyl stretch of MOC in SDS–water closely resembles that of MeOH, with two peaks appearing at approximately the same frequencies, despite the absence of an interface in the latter, while the corresponding CTC spectrum presents a single peak with a frequency ∼ 2 cm^–1^ lower than of the SDS–water system. These results support the preferential localization of MOC at the SDS–water interface rather than in the micelle core, an assignment that could not have been reached if only the MOC alkyne stretch had been used.

**Figure 5:**
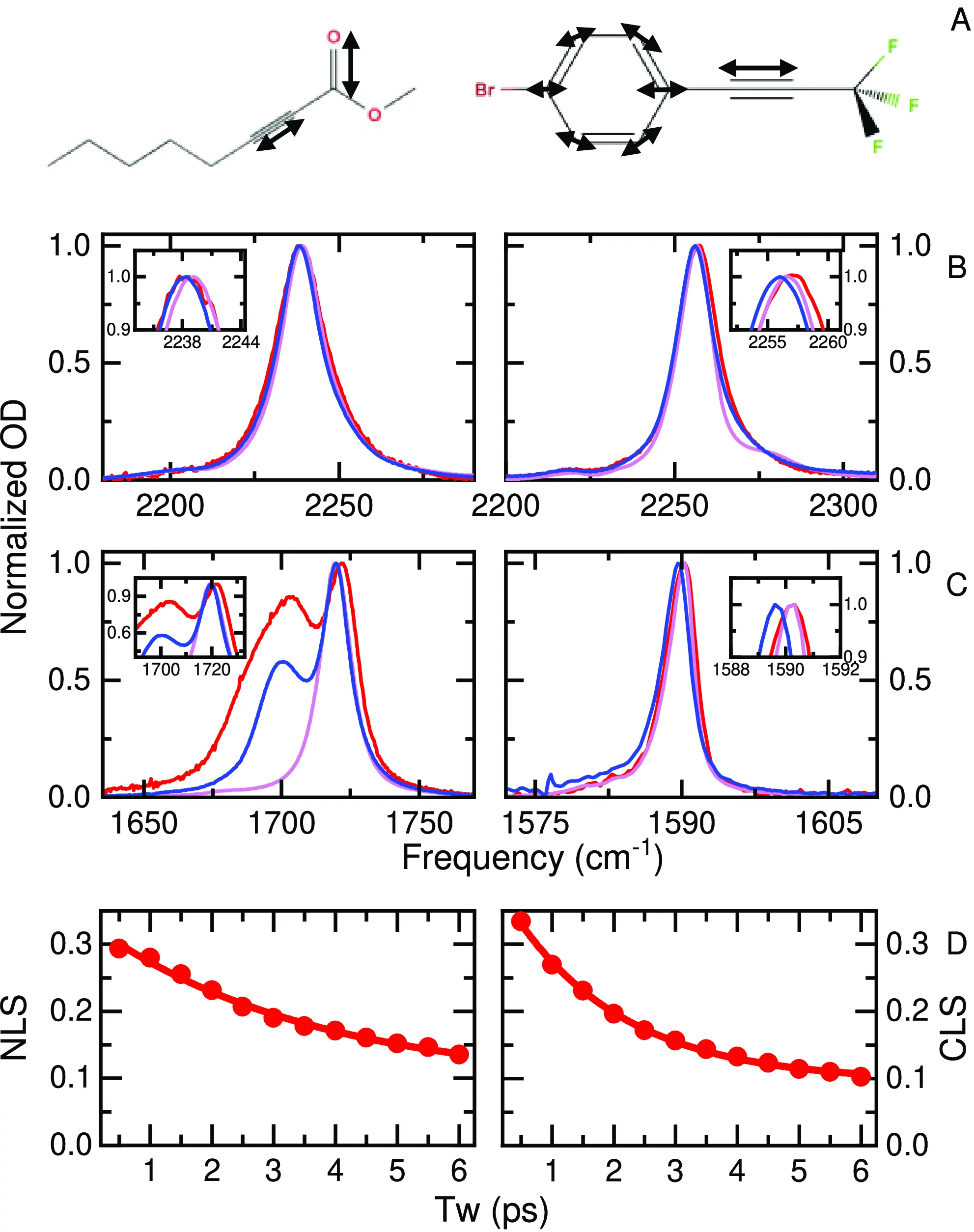
Panel A shows an illustration of the vibrational reporter modes for MOC (left) and TFPB (right). The normalized IR spectra for MOC and TFPB in SDS–water (red), MeOH (blue), and CTC (pink) are shown in panels B and C for the alkyne stretch and secondary modes (carbonyl stretch and ring breathing mode), respectively. Panel D shows the 2DIR dynamics for the alkyne stretch of the probes in the SDS micelle, extracted using the nodal line slope (NLS, left) for MOC and the central line slope (CLS, right) for TFPB.

The localization of TFPB in the SDS-micelle system is much simpler using IR solvatochromism. In this case, the alkyne stretch and the ring breathing modes exhibit single peaks with central frequencies of 2257 cm^–1^ and 1590 cm^–1^ with similar π* values corresponding to a nonpolar solvent between HEX and CTC. Further support for the location of the probe is obtained by comparing the central frequency of the TFPB ring breathing mode in the SDS micelles, CTC, and MeOH. In these cases, the central frequencies clearly indicate a chemical environment in the SDS-micelle closer to that of nonpolar CTC than to MeOH (Figure 5), verifying that TFPB is located in the hydrophobic core of the micelle (Figure 2 and Figure 5).

Besides the location obtained for both probes in the SDS–water environment, the alkyne stretch in both probes enables direct measurement of solvation dynamics in these two environments (i.e., interface and hydrophobic core) using 2DIR spectroscopy. While limited by the appearance of the water grating signal,^72^ spectral diffusion analysis on the 2DIR spectra without major distortions due to this signal (Figure S6) reveals that the alkyne stretch of MOC and TFPB have very distinct dynamics (Figure 5). An exponential model of the form $FFCF\left(T_{W}\right)=A_{1}e^{-t/\tau }+A_{2}$ for the spectral diffusion dynamics reveals characteristic times of ∼ 3.5 ps and ∼1.8 ps for alkyne stretches of MOC and TFPB, respectively (Table 1) and an unresolved dynamical component in the form of a constant (A_2_), which was previously shown to arise from long time dynamical components of the environment.^3^ This result shows that the micellar core probed by TFPB has faster dynamics than the interface. Many previous studies have shown that spectral diffusion dynamics at the micelles interface is slower than in the core,^6,39,73,74^ but these studies probed bulk water and the micelle interface or the interface of reverse micelle and confined water. However, a recent work using cyano-azido vibrational probes and 2DIR spectroscopy reported enhanced flexibility and molecular mobility center of the hydrophobic region of a bilayer when compared to intermediate zones.^6^ Hence, the dynamics reported by TFPB show that the SDS micellar core has a similar dynamics to that seen in lipid bilayers, where the interior is highly dynamic due to rapid structural fluctuations from the loose packing of the alkyl chain ends and the interface is more dynamically restricted due to the close packing of the alkyl groups.^6^ Furthermore, the alkyne stretch of MOC reports dynamics with a characteristic time of 3.5 ps when located at the interface compared to ∼ 2.3 ps in pure solvents or ∼ 3.0 in the more heterogeneous and constraint LA-NMA DES.^3^ This dynamics is in agreement with the probe being located at the interface where the crowded and restricted environment further slows its dynamics. This observation supports not only the idea of the MOC dynamics being controlled by an internal mode, but also its positioning at the interface since water-like environment should result in a dynamics close to 1 ps as previously described.^75^ At the crowded environment of the interface, the restricted location of the ester group allows the probe to sense the solvation dynamics. Overall, the results show that MOC is an effective dual IR probe for interrogating hydrophobic/hydrophilic interfaces and solvent environments due to its amphiphilic nature, while TFPB is an effective dual-probe for hydrophobic regions and so, each probe only interrogates regions with similar polarity.

**Table 1:** Fitting of Spectral Diffusion Data with a Single-Exponential Function for MOC and TFPB in the SDS-Micelle System

Probe	A_1_	τ (ps)	A_2_
MOC	0.24 ± 0.01	3.5 ± 0.5	0.09 ± 0.01
TFPB	0.31 ± 0.004	1.8 ± 0.1	0.096 ± 0.003

In summary, this work demonstrates that asymmetric alkynes are an excellent framework for the design of dual vibrational IR probes with tunable polarity. By combining the alkyne stretch with another vibrational reporter, the dual-probe approach enables to experimentally determine and characterize the probe location in complex systems. The utility of the alkyne-based dual-probe approach is demonstrated in two challenging heterogeneous systems: the LA-NMA DES and SDS micelles. In the first case, the MOC carbonyl stretch locates the probe within the NMA domain of the DES, corroborating previous results derived using only the alkyne stretch. In SDS micelles, MOC and TFPB reveal the expected preferential localization at the micelle interface and hydrophobic core, respectively, each having distinct solvation dynamics as demonstrated using 2DIR spectroscopy. Collectively, these findings highlight the applicability of the alkyne-based dual-probe strategy for studying structure, interactions, and dynamics in complex heterogeneous environments. Because both the polarity and the presence of a secondary reporter in the probe can be tuned independently through selective alkyne substitution, the present framework establishes a general strategy for designing vibrational probes targeted to specific heterogeneous environments, including designer solvents, biomolecular membranes, and soft materials.

## Supplementary Material



## Notes

Details on materials and methods: sample preparation, FTIR and 2DIR spectroscopy measurements. Additional tables containing: linear vibrational spectroscopy parameters, Kamlet–Taft and dielectric constant solvent parameters, and 2DIR spectroscopy fitting parameters. Vibrational spectroscopy: FTIR and 2DIR in DMSO, MeOH, and SDS-micelles. Correlations between central frequency of the alkyne stretches of MOC and TFPB versus Kamlet–Taft parameters and dielectric constant of each solvent (PDF)
