# Supplementary material for: A Dual Vibrational Probe Framework for Studying Solvation in Highly Heterogeneous Molecular Environments
Source: J Phys Chem Lett. 2026 Oct 14;17(43):12382–9. doi: 10.1021/acs.jpclett.6c02383 (PMC13726463; doi:10.1021/acs.jpclett.6c02383)
Supplement: Supplementary file 1 [file jz6c02383_si_001.pdf]

Supporting Information

A Dual Vibrational Probe Framework for Studying Solvation in Highly Heterogeneous Molecular Environments

Samadhan H. Deshmukh,† Laura X. Sepulveda-Montaño,† and Daniel G. Kuroda *

Department of Chemistry, Louisiana State University, Baton Rouge, Louisiana 70803, United States.

†Equal contribution in alphabetical order

*Address correspondence to dkuroda@lsu.edu

Materials and Methods

Sample preparation

All the R−C≡C−R′ (2-Octyne (OTY), 2-octyn-1-ol (OTO), 1-Chloro-2-octyne, Methyl octyne carbonate (MOC) and 1-bromo-4-(3,3,3-trifluoroprop-1-yn-1-yl) benzene (TFPB)) compounds ordered from Ambeed chemicals and used as it is. Sodium dodecyl sulfate (SDS) purchased from sigma- Aldrich (≥98.5%). All the solvents used were spectroscopic grade. DMSO (Sigma Aldrich, 99.9%), THF (Fisher, 99.9%), MeOH, Hexane, benzene, Toluene (Sigma Aldrich 99.8%), Dimethylformamide (DMF, 98% Sigma-Aldrich), carbon tetrachloride (CCl_4, Sigma Aldrich 99+%), Chloroform (CLF, Sigma Aldrich 99+%), propylene carbonate (PC, 99.5% Acros Organics) and D_2O (Sigma Aldrich 99.9%). 100 mM concentration of Alkyne as vibrational probe used for each sample. We prepared 0.5M SDS in H_2O/D_2O for SDS micelle sample as reported previously[1]. The solute was added after the preparation of SDS micelles.

Linear IR spectroscopy

FTIR spectra were recorded for each sample using a Bruker Tensor 27 spectrometer equipped with a liquid nitrogen-cooled mercury cadmium telluride (MCT) detector at a resolution of 0.5 cm^{-1}. Samples were held between two CaF_2 windows (2 mm thickness) separated by a 100 μm Teflon spacer to define the path length. Each spectrum was averaged over 40 scans to enhance the signal-to-noise ratio.

Non-linear 2DIR spectroscopy

The 2DIR experiments were conducted using a setup similar to that detailed in the literature.[2] A broadband IR pulse, ∼60 fs in temporal width, was generated using a Spectra-Physics Spitfire Ace Ti:Sapphire amplifier operating at a 5 kHz repetition rate, which was paired to an optical parametric amplifier (Spectra-Physics OPA-800C) which generates the signal (ω_1) and idler (ω_2). Finally, these passes through an AgGaS2 difference frequency generation (ω_1- ω_2) crystal where IR generated. The IR pulses were each split into three identical pulses having wavevectors k_1, k_2, and k_3, then focused onto the sample using a lens in a boxcar configuration. A photon echo (PE) was produced in the phase matching direction of $-k_1 + k_2 + k_3$ and heterodyned with a fourth pulse, the local oscillator (LO), that always preceded the PE by ∼0.6 ps. This combined signal was dispersed via a Triax Horiba monochromator with 100 grooves/mm and detected using a nitrogen-cooled mercury cadmium telluride (MCT) array detector from infrared systems development, consisting of 64 elements. Computer-controlled translational stages from PI Micos were used to control the time intervals between the pulses, represented as τ (pulses 1 and 2), the waiting time Tw (pulses 2 and 3), and t (pulses 3 and PE). The time interval Tw was scanned for 10 ps from 0 ps, with 1 ps time-steps. The time interval (τ) time varied from -2 to 2 ps, with a 5 fs time-step. Two Fourier transforms with respect to τ and t were utilized to convert the time domain signal S (τ, Tw, t) to the frequency domain S (ωτ, Tw, ωt). The 2DIR spectra of the C≡C stretch band of the

MOC and TFPB solutions were measured at room temperature with the same sample cell as used in the FTIR experiment.

Determination of the FFCF dynamics

The FFCF dynamics was determined using either nodal line slope (NLS) and center line slope (CLS) as these two metric report the same metric in the absence of noise.[3, 4]

Tables

Table S1. FTIR parameters for the two vibrational probe moieties of MOC: C≡C and C=O stretches in solvents and SDS micelles, determined using the first spectral moment, along with corresponding dielectric constants (Ɛ) and polarizability values (π*).

Solvents	C≡C stretch ω_0 (cm^{-1})	C=O stretch ω_0 (cm^{-1})	Solvent properties	
			Dielectric constant (Ɛ)	Polarizability (π*)[5]
HEX	2240.98	1726.75	1.88	-0.08
CTC	2239.04	1719.70	2.30	0.28
TOL	2237.40	1718.34	2.38	0.57
CLF	2237.14	1710.12	4.81	0.58
THF	2236.88	1718.36	7.58	0.58
BEN	2236.92	1717.12	2.27	0.59
MeOH	2238.42	1719.80	32.7	0.60
		1700.55		
DMF	2234.88	-*	36.7	0.88
DMSO	2233.82	1710.72	46.7	1.00
PC	2235.41	-*	64.9	0.83
SDS micelle	2238.66	1721.73	-	-
		1704.13		

* Note: the values for the carbonyl stretch of MOC in DMF and PC where not added as the C=O stretch band of the solvents overlaps with that of MOC and does not allow to perform proper background subtraction.

Table S2. FTIR parameters for the two vibrational probe moieties of TFPB: C≡C stretches and ring mode in solvents and SDS micelles, determined using the first spectral moment, along with corresponding dielectric constants (Ɛ) and polarizability values (π*).

Solvents	C≡C stretch ω_0 (cm^{-1})	Ring mode ω_0 (cm^{-1})	Solvent properties	
			Dielectric constant (Ɛ)	Polarizability (π*)[5]
HEX	2258.23	1591.05	1.88	-0.08
CTC	2256.63	1590.33	2.3	2.28
TOL	2254.82	1588.88	2.38	0.57
CLF	2257.06	1589.85	4.81	0.58
THF	2254.56	1588.64	7.58	0.58
BEN	2255.3	1588.88	2.27	0.59
MeOH	2256.02	1589.61	32.7	0.60
DMF	2255.21	1587.44	36.7	0.88
DMSO	2254.65	1587.19	46.7	1.00
PC	2256.95	1588.65	64.9	0.83
SDS micelle	2256.5	1590.33	-	-

Table S3. Fitting of spectral diffusion data with a single-exponential function for MOC in MeOH and DMSO.

Solvent	A	A_2	τ_c (ps)
MeOH	0.21 ± 0.01	0.05 ± 0.002	2.2 ± 0.1
DMSO	0.26 ± 0.003	0.04 ± 0.002	2.4 ± 0.1

Table S4. Fitting spectral diffusion data with a single-exponential function for TFPB in MeOH and DMSO.

Solvent	A	A_{inf}	τ_c (ps)
MeOH	0.30 ± 0.01	0.05 ± 0.003	1.6 ± 0.1
DMSO	0.41 ± 0.01	0.03 ± 0.01	3.0 ± 0.2

Table S5. Fitting of vibrational lifetime for MOC and TFPB in CTC, MeOH and SDS-H2O.

	A1	T1	A2	T2	<T10>
MOC/MeOH	0.25	4.40	0.75	0.65	1.60
MOC/CTC	0.25	3.80	0.75	0.59	1.39
MOC/SDS-H2O	0.23	5.44	0.77	0.69	1.78
MOC/DMSO	0.22	5.79	0.78	0.72	1.82
TFPB/MeOH	0.43	0.30	0.57	1.97	1.25
TFPB/SDS-H2O	0.33	0.79	0.67	2.78	2.11
TFPB/DMSO	0.76	0.36	0.24	2.55	0.89

Figures

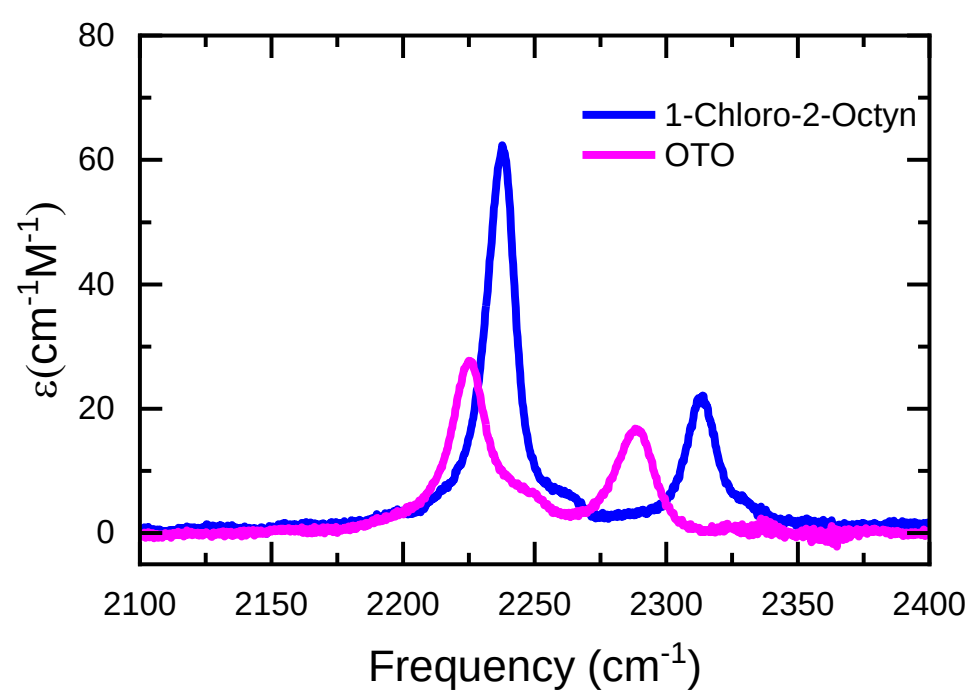

Figure S1. Molar extinction spectra of OTO (pink) and 1-Chloro-2-Octyn (blue) in CTC.

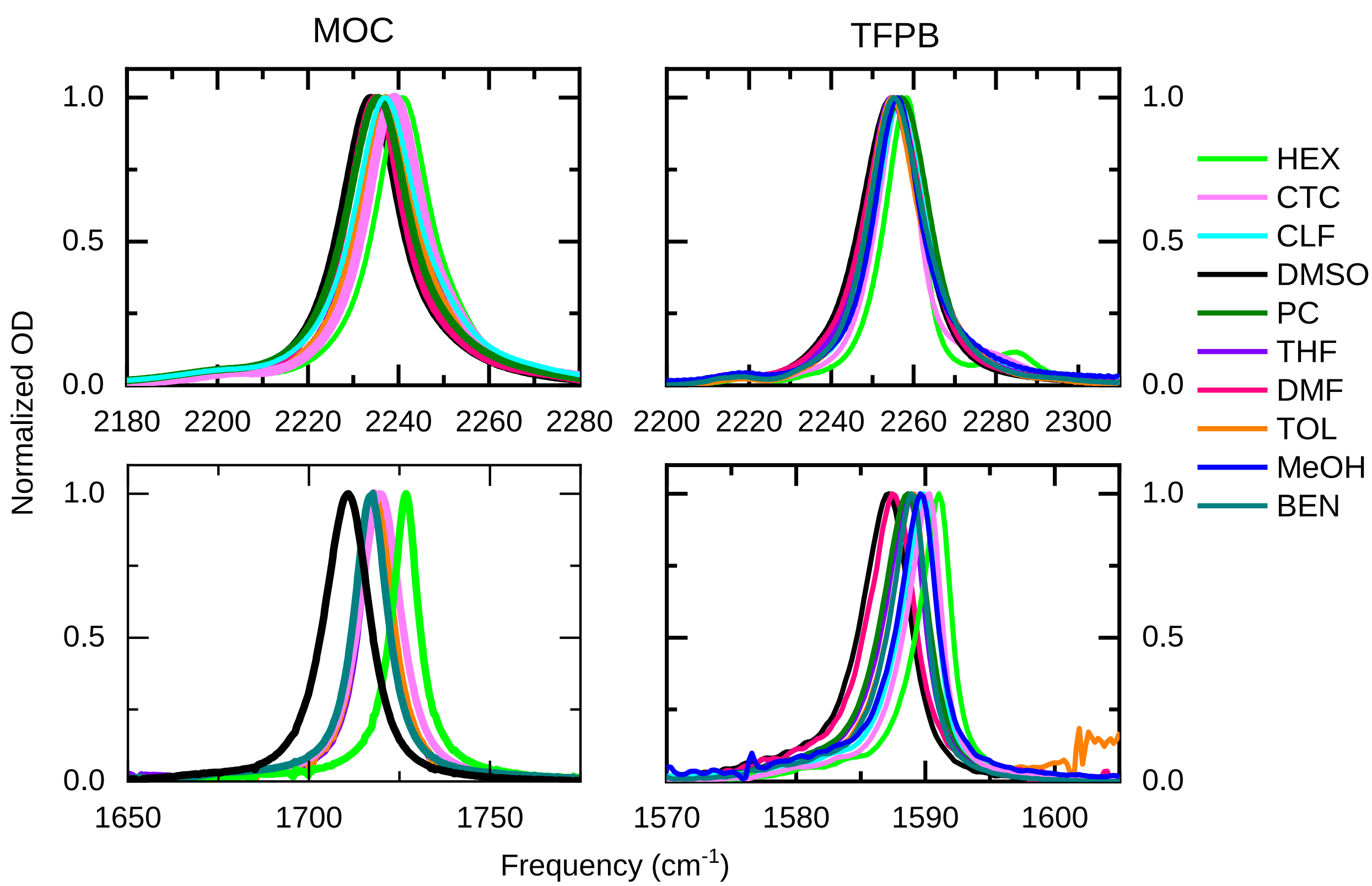

Figure S2. The first column shows the normalized FTIR spectra of MOC and the second for TFPB in various solvents, the top panel correspond to the C≡C stretch while the bottom panel shows the normalized carbonyl stretch of MOC and the ring mode of TFPB.

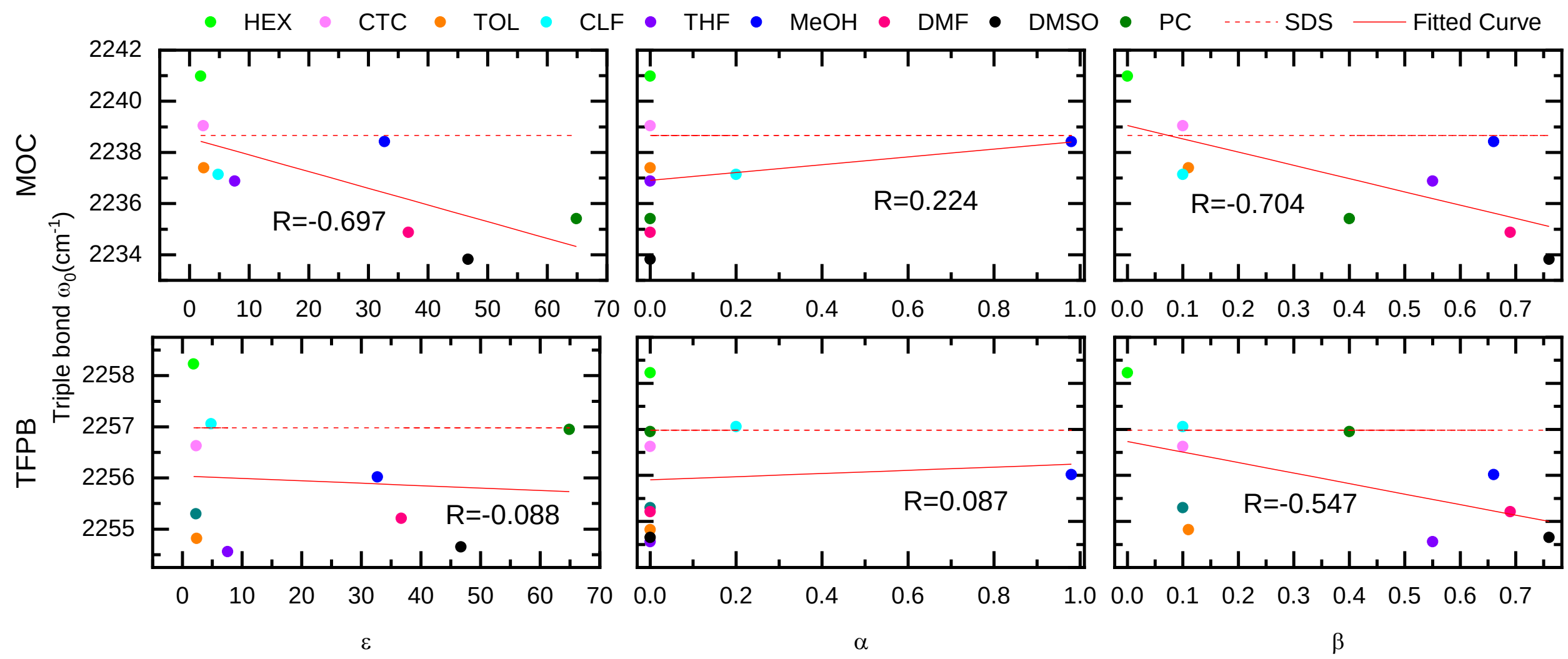

Figure S3. The C≡C stretching frequencies of MOC and TFPB in various solvents versus Dielectric constant (Ɛ) and Kamlet-Taft hydrogen-bond donating (α) and accepting (β) parameters.

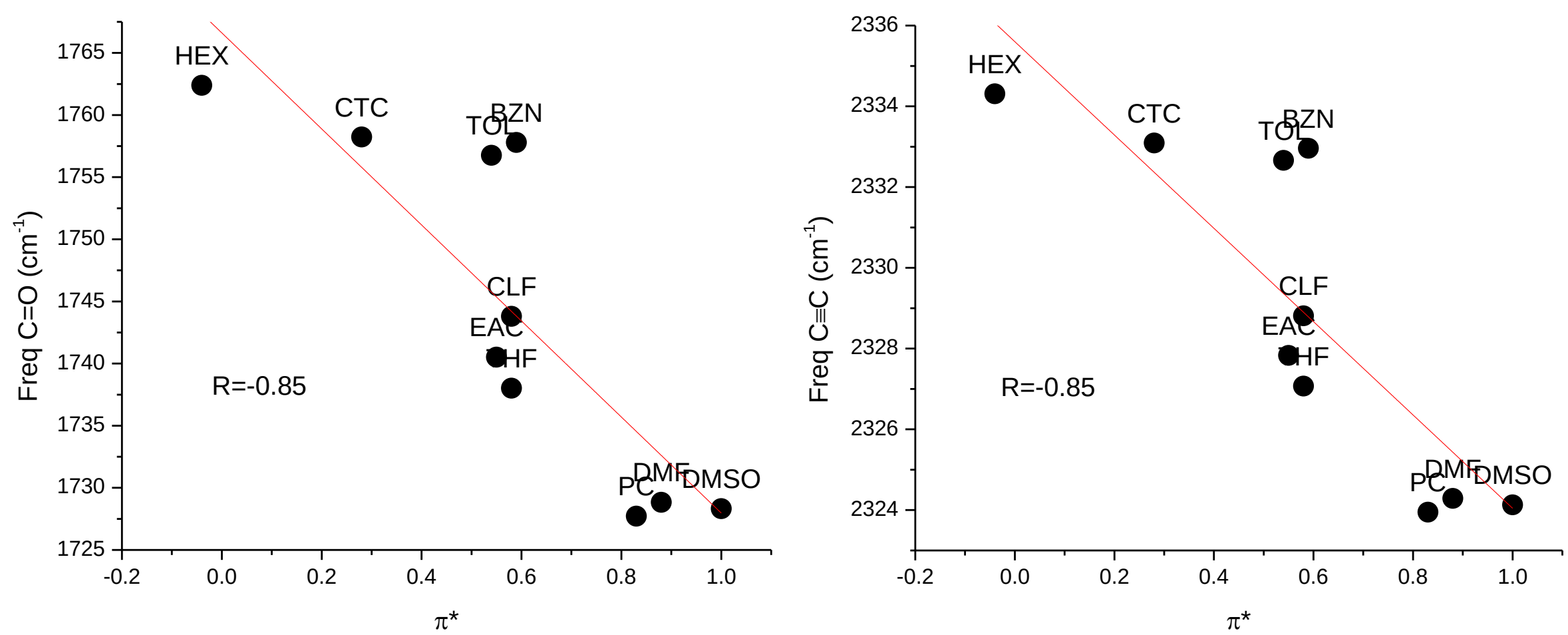

Figure S4. Frequencies of the carbonyl (left panel) and carbon triple bond (right panel) stretches determined from DFT computations using a B3LYP functional, the 6-31+g(d,p) basis set, and the corresponding polarizable continuum model (PCM).

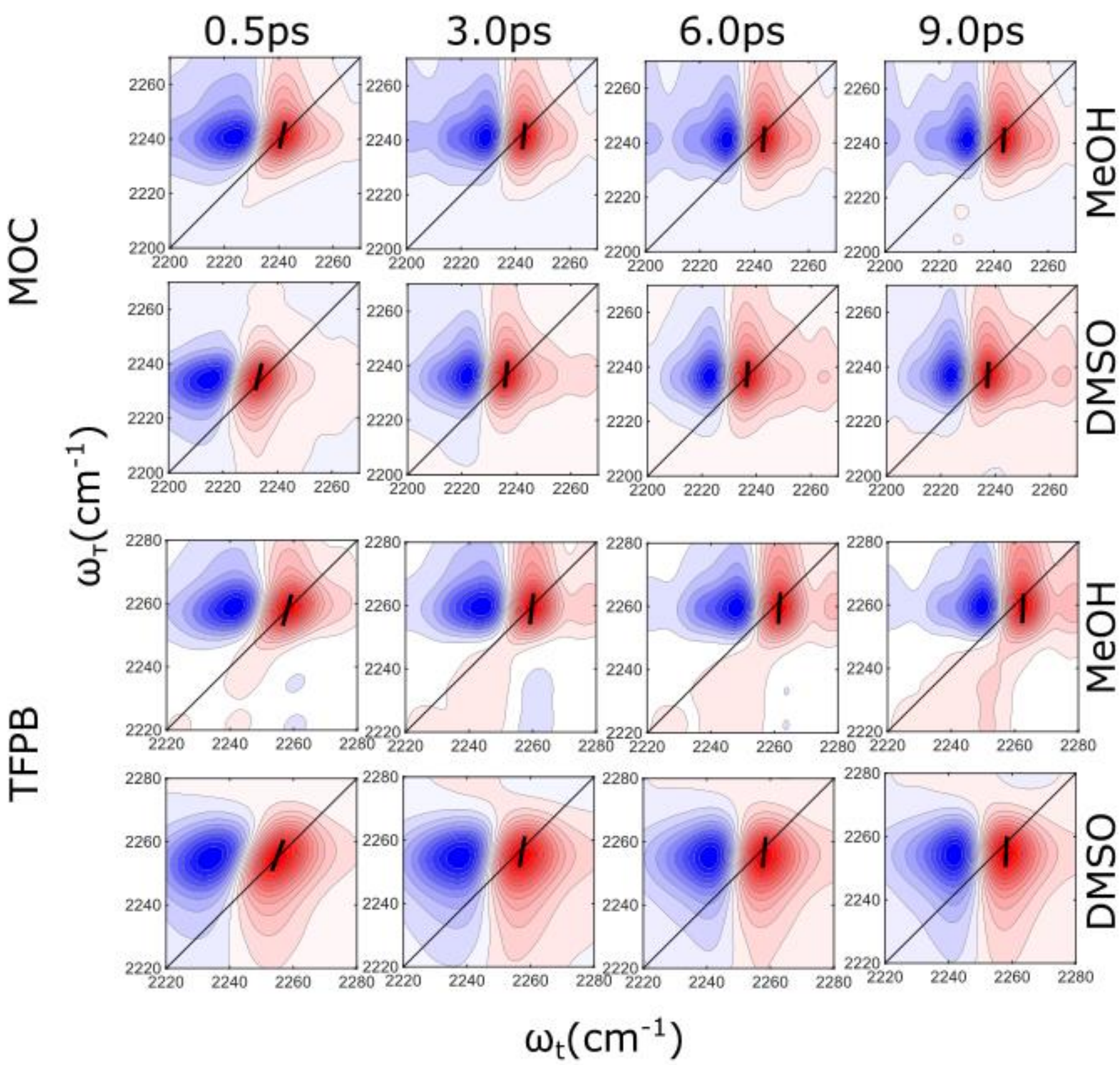

Figure S5. 2DIR spectra of MOC and TFPB in MeOH and DMSO.

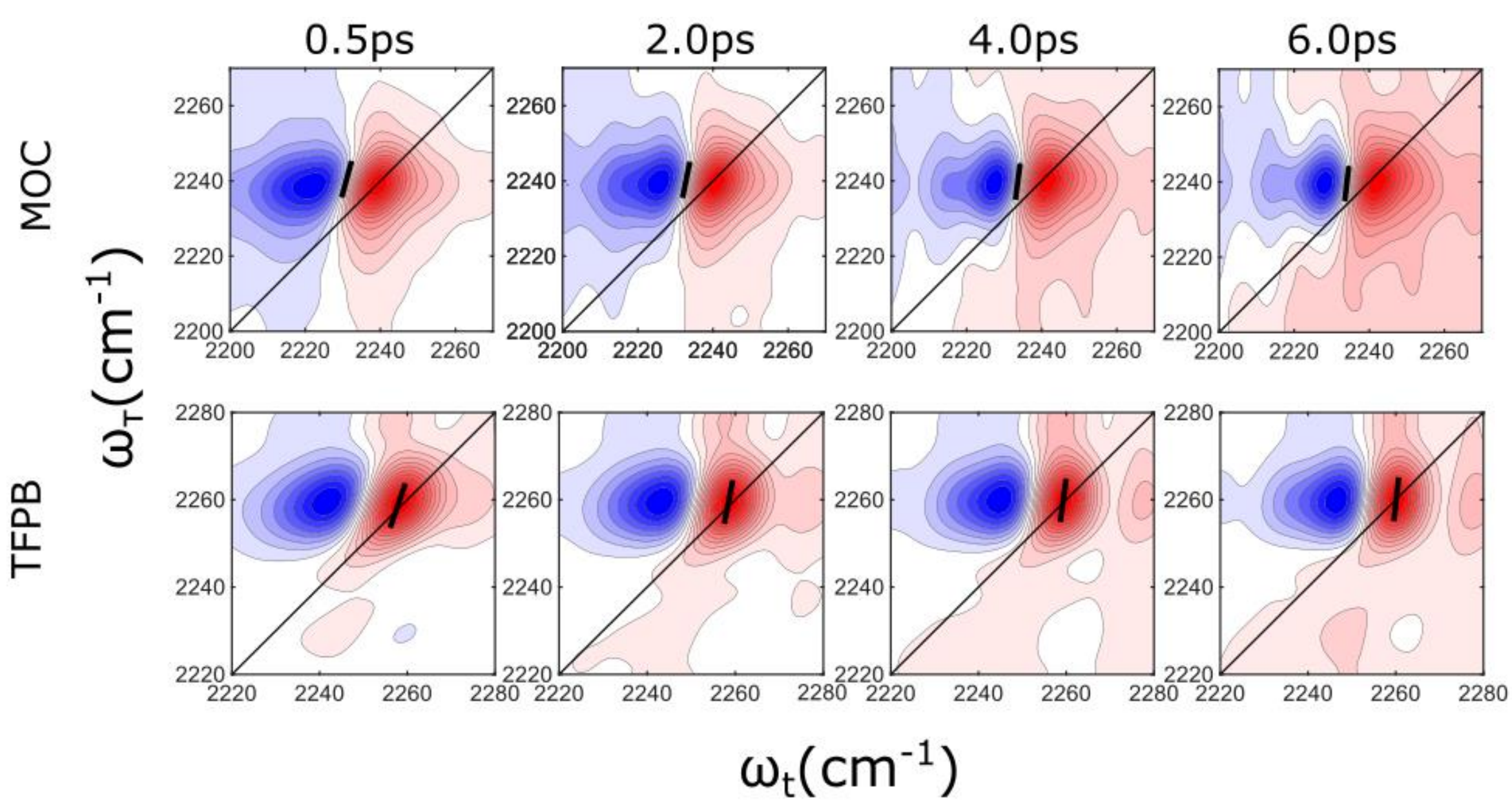

Figure S6. 2DIR spectra of MOC (top panel) and TFPB (bottom panel) in SDS micelle.

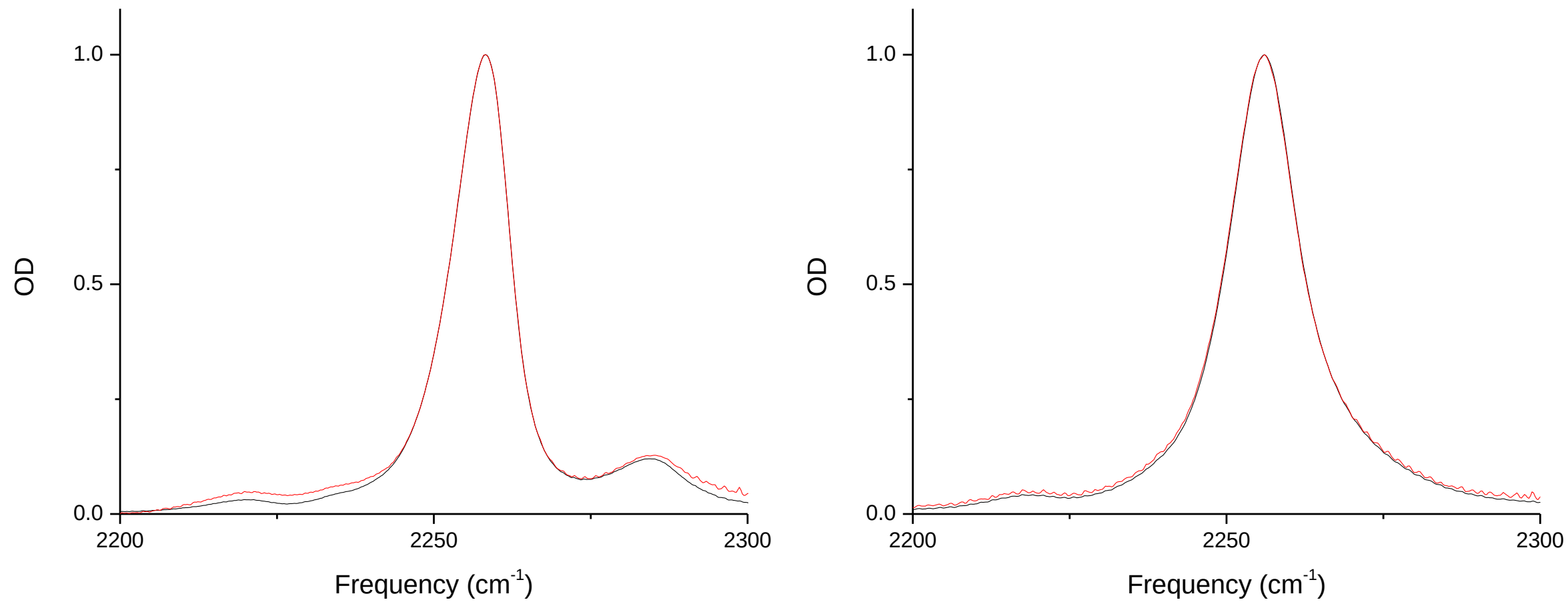

Figure S7. Normalized FTIR spectra of the TFPB C≡C stretch in hexane (left panel) and methanol (right panel) for 100mM (black) and 50 mM (red).

Equations

Molecule	Vibrational Probe	Solvatochromic equation
MOC	C≡C Stretch	$\omega_0 = -6.5 \pm 0.6 \cdot \pi^* + 2240.9 \pm 0.4$
	C=O Stretch	$\omega_0 = -15 \pm 4 \cdot \pi^* + 1725 \pm 2$
TFPB	Ring breathing	$\omega_0 = -3 \pm 1 \cdot \pi^* + 2257.4 \pm 0.7$
	C≡C Stretch	$\omega_0 = -3.6 \pm 0.5 \cdot \pi^* + 1591.1 \pm 0.4$

Equation S1. Solvatochromic equations for the dual probe molecules MOC and TBPB respect polarizability π^*.

References

(1) Volkov, V.; Hamm, P. A Two-Dimensional Infrared Study of Localization, Structure, and Dynamics of a Dipeptide in Membrane Environment. *Biophysical Journal* **2004**, *87*, 4213-4225. DOI: 10.1529/biophysj.104.045435.
(2) Fulfer, K. D.; Kuroda, D. G. Solvation Structure and Dynamics of the Lithium Ion in Organic Carbonate-Based Electrolytes: A Time-Dependent Infrared Spectroscopy Study. *The Journal of Physical Chemistry C* **2016**, *120*, 24011–24022. DOI: 10.1021/acs.jpcc.6b08607.
(3) Al-Mualem, Z. A.; Baiz, C. R. Generative Adversarial Neural Networks for Denoising Coherent Multidimensional Spectra. *The Journal of Physical Chemistry A* **2022**, *126* (23), 3816-3825. DOI: 10.1021/acs.jpca.2c02605.
(4) Gurung, A.; Kuroda, D. G. A new method based on pseudo-Zernike polynomials to analyze and extract dynamical and spectral information from the 2DIR spectra. *The Journal of Chemical Physics* **2023**, *159* (3), 034201. DOI: 10.1063/5.0154601.
(5) Kamlet, M. J.; Abboud, J. L. M.; Abraham, M. H.; Taft, R. Linear solvation energy relationships. 23. A comprehensive collection of the solvatochromic parameters,. pi.*,. alpha., and. beta., and some methods for simplifying the generalized solvatochromic equation. *The Journal of Organic Chemistry* **1983**, *48* (17), 2877-2887. DOI: 10.1021/jo00165a018.
